# Editorial: Rising stars in nutrition and inflammation

**DOI:** 10.3389/fnut.2023.1197351

**Published:** 2023-04-14

**Authors:** Honglin Dong, Yannan Jin

**Affiliations:** ^1^School of Health & Psychological Sciences, City, University of London, London, United Kingdom; ^2^Leicester School of Allied Health Sciences, De Montfort University, Leicester, United Kingdom

**Keywords:** inflammation, vitamin D, vitamin A, albumin to globulin ratio, extracellular vesicles, glutathione, high-fat diet, phytochemical

Inflammation occurs in acute and chronic disease states and interplays with one's nutritional status. Low-grade systemic inflammation has implications in the pathophysiology of age-related health issues and the major chronic diseases including cardiovascular disease, type 2 diabetes mellitus, Alzheimer's disease, and many types of cancer (1). High-fat diet (HFD) has shown to increase pro-inflammatory parameters including plasma high-sensitive C-reactive protein (hs-CRP), prostaglandin E_2_ (PGE_2_), thromboxane B_2_ (TXB_2_), and leukotriene B_4_ (LTB_4_) (2), whereas anti-inflammatory effects are reported among nutrients and phytochemicals such as fiber, unsaturated fatty acids, flavonoids, carotenoids, and certain dietary patterns (e.g, plant-based diet) through reducing hs-CRP, interleukin-6 (IL-6), and tumor necrosis factor alpha (TNF-α) and other biochemical markers of inflammation (3, 4).

The Research Topic aims to collect recent evidence and improve our knowledge and understanding on the research area in the nutrition and inflammation.

This collection includes eight papers.

The paper by Dai et al. using a fish model, demonstrated that HFD significantly increased TNF-α, IL-6, and monocyte chemoattractant protein-1 contents in the intestine, liver, and plasma. The authors further found HFD reduced the number of goblet cells, disturbed the mucin 2 secretion and thus LPS escapes the intestinal epithelial cells barrier via a transcellular pathway with the assistance of CD36.

Chen et al. conducted a retrospective analysis of 5,173 patients with acute ischemic stroke (AIS) at a hospital in China between 2013 and 2021. The patients with stroke-associated pneumonia (SAP, *n* = 897) had a significant lower albumin to globulin ratio (A/G) compared with non-SAP (*n* = 4,276; 1.12 ± 0.22 vs. 1.27 ± 0.23, *P* < 0.001). The patients with a lower A/G ( ≤ 1.09) had a higher risk of SAP (OR = 1.96, 95% CI, 1.56–2.46, *P* < 0.001) compared with patients with a A/G of 1.25–1.39. The authors indicated that appropriate measures to prevent SAP need to take for AIS patients with a low A/G and nutritional interventions to improve the A/G on the outcome of AIS and SAP should be investigated.

Dong et al. conducted a pilot randomized controlled trial investigating the influence of vitamin D supplementation on immune function of healthy aging people (55–85 years). The study showed vitamin D3 supplementation at 1,000 IU/day for 12 weeks significant increased plasma 25(OH)D levels compared with the control group, however, no effect was observed on phagocytic activity of granulocytes and monocytes, TNF, IL-6, and lymphocyte subsets apart from significantly decreased plasma creatinine concentrations, though 43% of the participants were vitamin D deficiency [25(OH)D < 25 nmol/L] at baseline. Higher dose of vitamin D supplementation might be needed for the future study.

Fan et al. reviewed the diagnostic use of the extracellular vesicles (EVs) in edible plants for different diseases such as neurodegenerative disease, and their therapeutic potentials for treating chronic diseases, such as stroke and Alzheimer's disease. Other novel aspects of EVs' application include their use as vehicles for safe drug delivery including genes and RNA drugs, due to their efficient transport through physiological barriers, such as the blood-brain barrier, and their high biocompatibility, low toxicity and immunogenicity. Research work continues in further exploring EVs' use as bioactive-substance delivery nanoplatforms and in anti-inflammatory treatments to meet the stringent demands of current clinical challenges.

Oxidative stress with systemic inflammation accounts for the development of age-related inflammatory diseases (inflammaging). Labarrere and Kassab in their review explored the mechanisms of glutathione (GSH) on its roles as an antioxidant in reducing the body's oxidation and inflammation associated with chronic inflammatory diseases and SARS-CoV-2 infection. A synergy of an adequate GSH redox status and 25-hydroxyvitamin D levels showed to further enhance immunity and diminish the adverse clinical consequences of COVID-19, particularly evident in African American communities. Further research is needed to investigate the efficacy of using GSH alone or together with other nutritional adjunctives on all types of inflammatory diseases.

Another antioxidant, epigallocatechin-3-gallate (EGCG), the main bioactive tea polyphenol was investigated in treating atopic dermatitis (AD) in an experiment with mice by Han et al. The authors formulated EGCG nanoparticles (EGCG-NPs) to investigate the bioavailability of EGCG to rescue cellular injury following the inhibition of necroptosis after AD. They found that EGCG-NPs elicited a significant amelioration of AD symptoms, a significant reduction in the expression of TNF-α, interferon-γ, interleukin-4 (IL-4), IL-6, and interleukin-17A, and inhibition of necroptosis rather than apoptosis in 2,4-dinitrochlorobenzene-induced AD. The authors concluded that EGCG-NPs represents a promising drug-delivery strategy for AD treatment by maintaining the Th1/Th2 balance and targeting necroptosis.

A review by Thirunavukarasu et al. discussed the roles of vitamin A in a wide range of physiological systems, particularly the significant roles in vision and T-cell mediated inflammation. A particular focus was given to the evidence on the nutritional treatment of age-related macular degeneration (AMD) and Stargardt Disease (STGD). The authors explored the key mechanisms which is proposed to be mediated by T-cells interacting with dietary vitamin A derivatives and the gut microbiome. The altered microbiome has been observed in AMD and STGD patients, leading to an emerging direction of research in the interactions of vitamin A and pro-vitamin A with the gut-eye immunological axis.

Jin and Arroo explored the *in vivo* evidence on the protective effects of flavonoids and carotenoids against diabetic complications. An overview of the recent preclinical and clinical data showed the potentials of both groups of phytochemicals for further development into novel drugs/therapies for treating diabetes and its complications. Yet their bioavailability, safety and tolerability need to be further investigated via long-term randomized controlled trials. Such evidence is warranted before confident clinical applications.

The above results represent new relevant data on nutrition and inflammation. We expect this research collection will trigger more original research in this important research area to further explore the anti-inflammatory potentials of nutrients, polyphenols and dietary patterns.

## Author contributions

All authors listed have made a substantial, direct, and intellectual contribution to the work and approved it for publication.
